# Supplementary material for: Vortex states in an acoustic Weyl crystal with a topological lattice defect
Source: Nat Commun. 2021 Jun 16;12:3654. doi: 10.1038/s41467-021-23963-7 (PMC8209201; doi:10.1038/s41467-021-23963-7)
Supplement: Supplementary file 1 — Supplementary Information [file 41467_2021_23963_MOESM1_ESM.pdf]

Supplementary Information for

"Vortex states in an acoustic Weyl crystal with a topological lattice defect"

Q. Wang, Y. Ge, H.-X. Sun, H. Xue, D. Jia, Y.-J. Guan, S.-Q Yuan, B. Zhang, and Y. D. Chong

SUPPLEMENTARY NOTE 1. NUMERICAL SIMULATION FOR THE CHARGE OF WEYL POINTS

In this section, we describe the numerical determination of the charges of the Weyl points for the 3D acoustic structure in the absence of a topological lattice defect (TLD). We adopt the method described by Soluyanov *et al.* [1]. A spherical surface is chosen to enclose one Weyl point, and we calculate the Berry phase around loops of constant latitudinal angle θ, as shown in Fig. 1**a**. The evolution of the Berry phase as a function of θ is plotted in Fig. 1**b**,**c** for the Weyl points at K and H respectively. The Berry phase for the lower bands (red circles) exhibits a 2π (-2π) change, implying that the charge for the two Weyl points are +1 and -1. The eigenfunctions used to calculate the Berry phase are obtained from *ab initio* acoustic simulations performed with Comsol Multiphysics.

SUPPLEMENTARY NOTE 2. TLD-BOUND MODES IN A CONTINUUM WEYL MODEL

In the vicinity of a Weyl point, the wavefunctions are governed by an effective long-wavelength Weyl Hamiltonian

$$\mathcal{H}_0 = -i\big(\tau_z\sigma_x\partial_x + \sigma_y\partial_y\big) + m(k_z)\tau_z\sigma_z, \tag{S1}$$

where τ_i (σ_i) denote valley (sublattice) Pauli matrices, and $m(k_z)$ is some function of k_z. Here we rescale the Fermi velocity to 1. When $k_z \to 0(\pi)$, $m(k_z) \to +(-)k_z$, which indicates the Weyl nodes at K and K' (H and H') have the same chirality. As the system preserves time reversal symmetry, $m(k_z) = -m(-k_z)$. For convenience, here we suppose that $m(k_z) > 0$ for $k_z > 0$.

As described in the main text, the TLD is introduced by a "cut-and-glue" procedure in which a $\pi/3$ segment is cut from the triangular lattice in the x-y-plane, and the edges are

rejoined. Ruegg and Lin have previously studied this problem in the context of graphene [2], and the key steps of their analysis are summarised here for convenience.

The effects of the "cut-and-glue" procedure are modeled as a boundary condition along the edges of the cut. From a visual inspection of the lattice, one can deduce that crossing the cut swaps both the valley and sublattice indices. The boundary condition turns out to have the specific form [2, 3]

$$\Psi_L = \begin{pmatrix} 0 & 0 & 0 & e^{i\eta} \\ 0 & 0 & e^{i\eta^*} & 0 \\ 0 & e^{i\eta^*} & 0 & 0 \\ e^{i\eta} & 0 & 0 & 0 \end{pmatrix} \Psi_U, \tag{S2}$$

where $\eta = 2\pi/3$, and Ψ_L and Ψ_U indicate the wave function for the upper and lower side of the cut, which take the form

$$\Psi_{L,U} = \begin{pmatrix} \Psi_{+A} \\ \Psi_{+B} \\ \Psi_{-A} \\ \Psi_{-B} \end{pmatrix}_{L,U} \tag{S3}$$

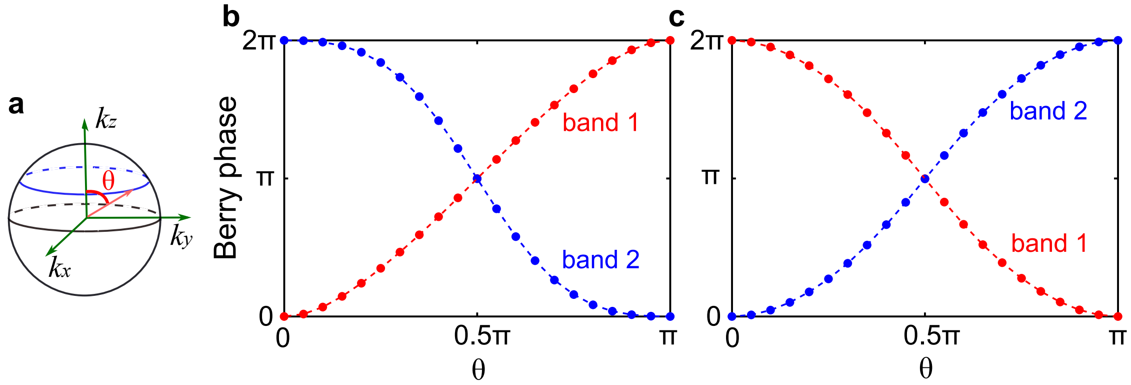

SUPPLEMENTARY FIGURE 1. Numerical calculation of Weyl point charges. **a**, Definition of the sphere in momentum space enclosing the Weyl points, and the latitudinal angle θ. Here, $k_{x,y}$ are given in units of $2\pi/(\sqrt{3}a)$ and k_z is in units of $2\pi/L$ (where a is the unit cell side length and L is the periodicity along z, as defined in Fig. 1 of the main text). For the numerical calculations, the sphere radius is set to $r = 0.2$. **b**,**c**, Calculated evolution of the Berry phase versus θ for spheres enclosing the Weyl points at K (**b**) and H (**c**). Blue and red indicate the lower and upper bands respectively.

where $\Psi_{\pm A/B}(r) \equiv \langle r|K_\pm, A/B\rangle$ and $|K_\pm, A/B\rangle$ denote a set of Weyl point states with $\pm$ indexing the Weyl nodes at K and K' (or H and H'), and A/B indexing the sublattice.

To deal with this boundary condition, we introduce polar coordinates (r, θ) defined in the original (undistorted) space, with $\theta \in [0, 5\pi/3]$. Then we introduce the gauge transformation

$$H = (SVU)H_0(SVU)^\dagger, \quad \text{where} \begin{cases} U_\theta &= e^{i\theta\sigma_z\tau_z/2} \\ V_\theta &= e^{i\theta\sigma_y\tau_y/4\Omega} \\ S &= (1 + i\tau_x\sigma_y), \\ \Omega &= 5/6. \end{cases} \tag{S4}$$

In polar coordinates, the transformed Hamiltonian is

$$\mathcal{H}_{\tau'} = \frac{-i}{r}\left[\left(r\partial_r + \frac{1}{2}\right)\tau'\sigma_x + \left(\partial_\theta + i\tau'\frac{1}{4\Omega}\right)\sigma_y\right] + m(k_z)\tau'\sigma_z. \tag{S5}$$

Here, $\tau' = \pm 1$ is a pseudospin index for the two blocks in the transformed Hamiltonian. The $i(1/4\Omega)\tau'$ term is introduced by the presence of the TLD, and takes the form of a pseudo-magnetic vector potential $\mathbf{A} = (4\Omega r)^{-1}\mathbf{e}_\theta$, corresponding to a singular magnetic flux localised at the origin.

The transformed eigenfunction $\Psi(r, \theta)$ obeys the boundary condition

$$\Psi(r,\ \theta = 5\pi/3) = -\Psi(r, \theta = 0). \tag{S6}$$

We now define the scaled polar angle $\varphi = \theta/\Omega$, where $\varphi \in [0, 2\pi]$. The rescaled azimuthal derivative is $\partial_\varphi = \partial_\theta/\Omega$. The boundary condition (S6) now becomes

$$\Psi(r,\ \varphi = 2\pi) = -\Psi(r, \varphi = 0). \tag{S7}$$

One can then search for localised solutions by taking a central region of radius $r < \rho$ to be a trivial insulator [2, 4, 5]. For each k_z, there is a single solution arising from one of the two choices of τ' ($\tau' = 1$ for $0 < k_z < \pi/L$, and $\tau' = -1$ for $-\pi/L < k_z < 0$)[2]; as the system preserve time reversal symmetry, the defect mode for $-k_z$ should be the counterpart of solution at k_z with the same energy but the opposite chirality.

Another way to deduce the existence of the localised TLD-bound modes is to track the evolution of the Chern insulator edge states as the lattice is put together. Here, we extend an argument from Ref. [2] to show that the existence of an in-gap defect mode should be insensitive to the details of the lattice configuration near the TLD.

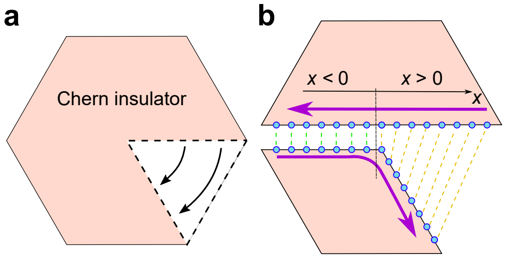

SUPPLEMENTARY FIGURE 2. Construction of a Chern insulator with a TLD. **a**, Cut-and-glue procedure, based on deleting a wedge and reattaching the edges. **b**, Procedure based on connecting two differently-shaped semi-infinite domains by adding bonds between the different edges (green and yellow dashes).

As shown in Fig. 2**a**, we are interested in a 2D system formed by a "cut-and-glue" construction: a $\pi/6$ wedge is deleted from a Chern insulator, and the edges of the wedges are reattached. Alternatively, such a system can be assembled as shown in Fig. 2**b**: we start with two differently-shaped Chern insulator domains (top and bottom orange regions), and then introduce bonds connecting the different edges (green and yellow dashes in Fig. 2**b**).

Prior to the connection, the bulk-edge correspondence principle implies that each domain supports one-way edge states along their boundaries (magenta arrows in Fig. 2**b**). When the connection is established, the edge states couple to each other. We can treat the parts of the edge far to the left of the TLD ($x \ll 0$ in Fig. 2**b**) and far to the right of the TLD ($x \gg 0$ in Fig. 2**b**) separately. Modeling these parts by L and R respectively, we introduce the edge state Hamiltonians

$$\mathcal{H}_{L,R} = k_x \sigma_z + t \cos\phi_{L,R}\, \sigma_x + t \sin\phi_{L,R}\, \sigma_y. \tag{S8}$$

Here, k_x is the momentum along the edge, which is a good quantum number far from the TLD; σ_i ($i = x, y, z$) are the Pauli matrices, with spinor components representing the two counterpropagating edge states; and t and $\phi_{L,R}$ parameterise an arbitrary coupling between the edge states introduced by the connection. The phase $\phi_{L,R}$ need not be the same on the two parts of the edge, due to the kink (60° bend) on the lower domain and other local perturbations associated with the central region of the TLD.

In the position representation, Eq. (S8) supports bound solutions of the form

$$\psi_L(x) \propto \; e^{\sqrt{t^2-E^2}x} \begin{pmatrix} te^{-i\phi_L} \\ E + i\sqrt{t^2-E^2} \end{pmatrix}, \tag{S9}$$

$$\psi_R(x) \propto \; e^{-\sqrt{t^2-E^2}x} \begin{pmatrix} te^{-i\phi_R} \\ E - i\sqrt{t^2-E^2} \end{pmatrix}. \tag{S10}$$

Now consider continuity relations of the form $\psi_L(0) = e^{i\varphi}\,\psi_R(0)$, where φ is an arbitrary phase. A bound state exists provided

$$E = t\cos\left(\frac{\phi_L - \phi_R}{2}\right). \tag{S11}$$

Thus there is a single bound state solution, whose energy ranges from $-t$ to t.

SUPPLEMENTARY NOTE 3. EFFECTS OF DISORDER ON TLD-BOUND MODES

As discussed in the previous section, the TLD-bound modes can be mapped to defect modes of 2D Chern insulators. This implies that they should be robust against in-plane disorder. To investigate this, we performed numerical simulations of acoustic structures with varying amounts of disorder. The disorder is implemented by setting the diameter d of each solid rod passing through the central air sheet as $d = D_0 - \Delta \cdot \text{rand}(1)$, where $D_0 = 1.6\,\text{cm}$ is the rod diameter in the disorder-free case, Δ parameterises the disorder strength, and rand(1) is drawn independently from a uniform random distribution over $(0,1)$. The structure depicted in Fig. 3**a** was generated with $\Delta = 1.2\,\text{cm}$ (i.e., rod diameters between 0.4 cm and 1.6 cm). The disorder is assumed to be entirely in-plane, meaning that different layers of the 3D structure have identical disorder configurations.

In Fig. 3**b**, the eigenfrequencies for the $k_z = \pi/2L$ modes are plotted versus the disorder strength Δ. With increasing disorder, the bulk gap shrinks, as expected. The TLD-bound mode survives up to very strong disorder ($\Delta \approx 1.4$cm) where the gap is almost closed.

Fig. 3**c** shows the the acoustic pressure distribution calculated for the TLD-bound modes at $\Delta = 1.2$cm and $k_z = \pi/2L$. (For comparison, refer to the results for the disorder-free structure in Fig. 2**c** of the main text.) From the intensity and phase distributions, we see that the mode continues to be localised near the center of the TLD, and that it has OAM of $+1$, unchanged from the disorder-free case.

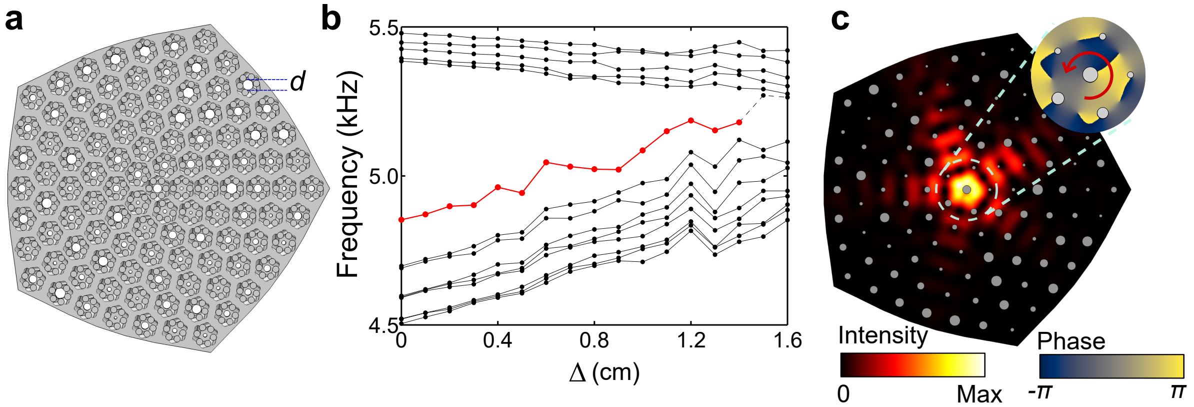

SUPPLEMENTARY FIGURE 3. Effect of disorder on TLD-bound modes. **a**, Cross-sectional schematic of a disordered structure. Each solid rod in each unit cell is assigned diameter d $\in [D_0, D_0-\Delta]$ where $D_0 = 1.6\,\mathrm{cm}$ and Δ is a disorder strength parameter. This structure is generated with $\Delta = 1.2\,\mathrm{cm}$. All other parameters are the same as in the ordered structure described in the main text. **b**, Calculated eigenfrequenies at $k_z = \pi/2L$ for different values of Δ. Red (black) circles indicate the TLD-bound modes (bulk modes). The random rod diameters are re-drawn for each Δ. **c**, Simulated in-plane acoustic pressure intensity distribution for $\Delta = 1.2\,\mathrm{cm}$ and $k_z = \pi/2L$, for z located at the midpoint of the central air sheet. Gray circles are the solid rods with different diameters. Inset: phase distribution near the TLD, showing that the TLD-bound modes retains its winding number of +1.

SUPPLEMENTARY NOTE 4. FIELD DISTRIBUTION MEASUREMENTS

In the main text, we showed the measured field distribution for CW and CCW vortex excitation at 5.6 kHz, a frequency that supports TLD-bound modes (Fig. 4**c**–h). For comparison, Fig. 4 shows the measured field distributions at 4.2 kHz and 6.0 kHz, frequencies outside the range where TLD-bound modes are predicted to exist. In these two cases, neither vortex produces a localised hot spot near the center of the TLD.

Fig. 5 shows the source and probe positions inside the sample layers for the various experiments. The probe positions used for field mapping (Fig. 3**d**–**f** and Fig. 4**c**,**d** of the main text) are shown as red circles. When exciting close to the defect (Fig. 3**a** and Fig. 3**c**–**f** of the main text), the source is located at the blue star in the bottom layer of the sample. When exciting the bulk, the source is located at the magenta star in the bottom layer. When averaging the spectra over points close to the TLD (Fig. 3**a** and Fig. 4**b** of the main text),

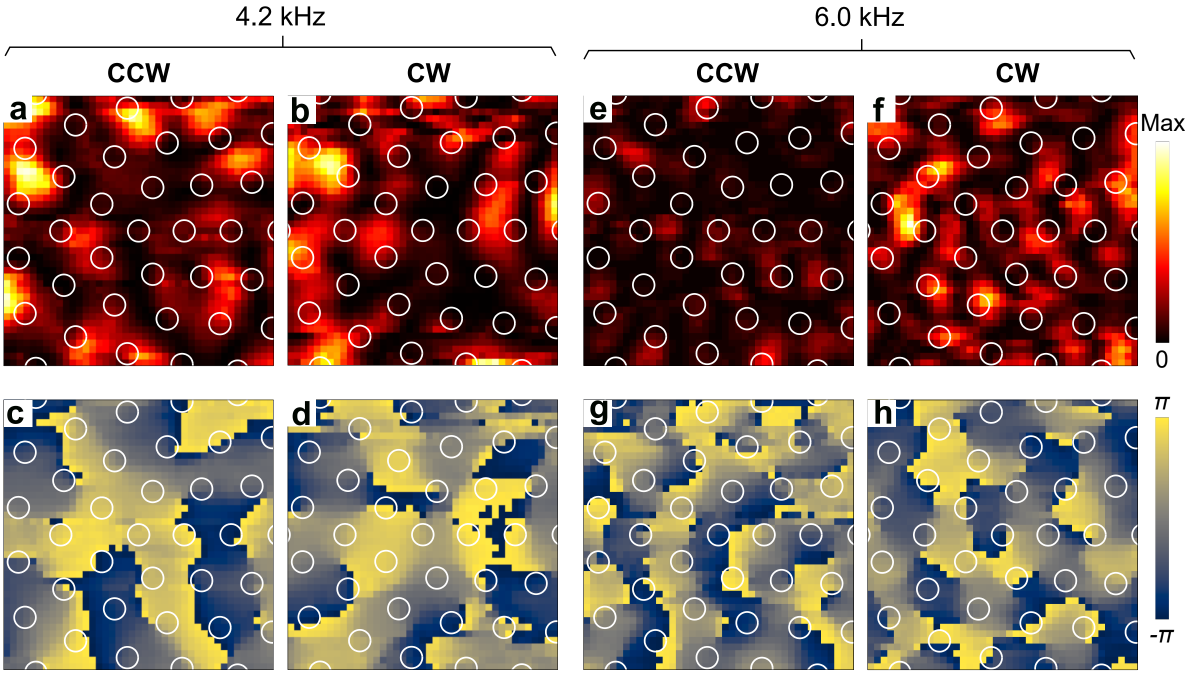

SUPPLEMENTARY FIGURE 4. Measured field distributions for vortex excitation at frequencies with no TLD-bound modes. **a**–**d**, Measured intensity distributions (**a**, **b**) and phase distributions (**c**, **d**) for CCW and CW vortex excitations at $f = 4.2\,\mathrm{kHz}$. **e**–**h**, Measured intensity distributions (**e**, **f**) and phase distributions (**g**, **h**) for CCW and CW vortex excitations at $f = 6.0\,\mathrm{kHz}$.

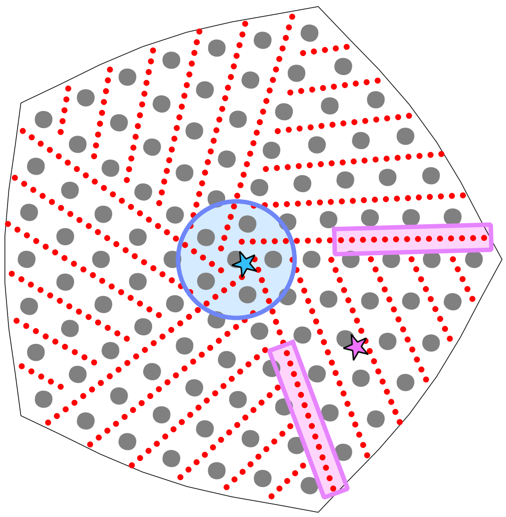

SUPPLEMENTARY FIGURE 5. Probe and source positions in each sample layer. Red circles indicate the probe positions for field-mapping measurements. Blue and magenta stars indicate the position of the source for defect and bulk excitation, respectively. Blue circle and magenta rectangles indicate the measurement points used to plot the defect and bulk spectra, respectively.

we use the points enclosed in the blue circle. For bulk spectra (Fig. 3**b** of the main text), we average the intensities over the points in the magenta rectangles, away from the TLD.

[1] Alexey A Soluyanov, Dominik Gresch, Zhijun Wang, QuanSheng Wu, Matthias Troyer, Xi Dai, and B Andrei Bernevig, "Type-ii weyl semimetals," Nature **527**, 495–498 (2015).

[2] Andreas Rüegg and Chungwei Lin, "Bound states of conical singularities in graphene-based topological insulators," Phys. Rev. Lett. **110**, 046401 (2013).

[3] Qiang Wang, Haoran Xue, Baile Zhang, and Y. D. Chong, "Observation of protected photonic edge states induced by real-space topological lattice defects," Phys. Rev. Lett. **124**, 243602 (2020).

[4] Nicholas Read and Dmitry Green, "Paired states of fermions in two dimensions with breaking of parity and time-reversal symmetries and the fractional quantum hall effect," Phys. Rev. B **61**, 10267 (2000).

[5] Abhishek Roy and Michael Stone, "Fullerenes, zero-modes and self-adjoint extensions," J. Phys. A: Math. Theor. **43**, 015203 (2009).
